# Verification of reprojected planar images generated from a ring-configured cadmium zinc telluride gamma camera in scintigraphy for diagnosing transthyretin cardiac amyloidosis

**DOI:** 10.1093/ehjimp/qyae051

**Published:** 2024-05-31

**Authors:** Irma Cerić Andelius, Ragnheidur Fridriksdóttir, David Minarik, Fredrik Hedeer, Anna Stenvall, Elin Trägårdh, Jenny Oddstig

**Affiliations:** Radiation Physics, Department of Haematology, Oncology and Radiation Physicis, Skåne University Hospital, 221 85 Lund, Sweden; Department of Translational Medicine and Wallenberg Centre of Molecular Medicine, Lund University, Carl Bertil Laurells gata 9, 205 02 Malmö, Sweden; Department of Translational Medicine and Wallenberg Centre of Molecular Medicine, Lund University, Carl Bertil Laurells gata 9, 205 02 Malmö, Sweden; Department of Clinical Physiology and Nuclear Medicine, Skåne University Hospital, Entrégatan 7, 221 85 Lund/Inga Marie Nilssons gata 47, 205 02 Malmö, Sweden; Radiation Physics, Department of Haematology, Oncology and Radiation Physicis, Skåne University Hospital, 221 85 Lund, Sweden; Department of Translational Medicine and Wallenberg Centre of Molecular Medicine, Lund University, Carl Bertil Laurells gata 9, 205 02 Malmö, Sweden; Department of Clinical Physiology and Nuclear Medicine, Skåne University Hospital, Entrégatan 7, 221 85 Lund/Inga Marie Nilssons gata 47, 205 02 Malmö, Sweden; Department of Clinical Sciences, Lund University, Sölvegatan 19, 221 84 Lund, Sweden; Radiation Physics, Department of Haematology, Oncology and Radiation Physicis, Skåne University Hospital, 221 85 Lund, Sweden; Department of Translational Medicine and Wallenberg Centre of Molecular Medicine, Lund University, Carl Bertil Laurells gata 9, 205 02 Malmö, Sweden; Department of Clinical Physiology and Nuclear Medicine, Skåne University Hospital, Entrégatan 7, 221 85 Lund/Inga Marie Nilssons gata 47, 205 02 Malmö, Sweden; Radiation Physics, Department of Haematology, Oncology and Radiation Physicis, Skåne University Hospital, 221 85 Lund, Sweden; Department of Translational Medicine and Wallenberg Centre of Molecular Medicine, Lund University, Carl Bertil Laurells gata 9, 205 02 Malmö, Sweden

**Keywords:** reprojected planar images, ring-configured, StarGuide, Perugini score, cardiac amyloidosis, CZT

## Abstract

**Aims:**

Non-invasive diagnosis of amyloid transthyretin (ATTR) cardiac amyloidosis using planar scintigraphy and single-photon emission computed tomography-computed tomography (SPECT-CT) with [^99m^Tc]Tc-3,3-diphosphono-1,2-propanodicarboxylic acid ([^99m^Tc]Tc-DPD) has high specificity and sensitivity. However, the introduction of ring-configured cadmium zinc telluride (CZT) gamma cameras warrants an update in the acquisition method since these systems are not able to perform planar scintigraphy. We aimed to verify the use of reprojected planar images from SPECT-CT as a replacement for planar scintigraphy in evaluating ATTR-amyloidosis.

**Methods and results:**

The study examined 30 patients referred for clinically indicated [^99m^Tc]Tc-DPD scintigraphy who were scanned with both a conventional gamma camera and a ring-configured CZT gamma camera. Planar scintigraphy from the conventional gamma camera was compared with reprojected planar images from the ring-configured CZT gamma camera. The images were evaluated in regard to image quality and Perugini visual score in a blinded fashion by three nuclear medicine physicians. Heart-to-contralateral (H/CL) ratios were calculated. There were 27 patients who had an identical Perugini score in planar and reprojected planar images, yielding a strong level of agreement and inter-rater reliability among the three readers. The H/CL ratios showed a strong correlation ratio (*r* = 0.98, *P* < 0.0001). A shift towards lower image quality was seen for the reprojected images.

**Conclusion:**

Reprojected planar images generated from a ring-configured CZT gamma camera combined with SPECT-CT can be used to score ATTR amyloidosis and extract H/CL ratios in the same way as planar images and SPECT-CT from a conventional gamma camera.

## Graphical Abstract

**Graphical Abstract:**
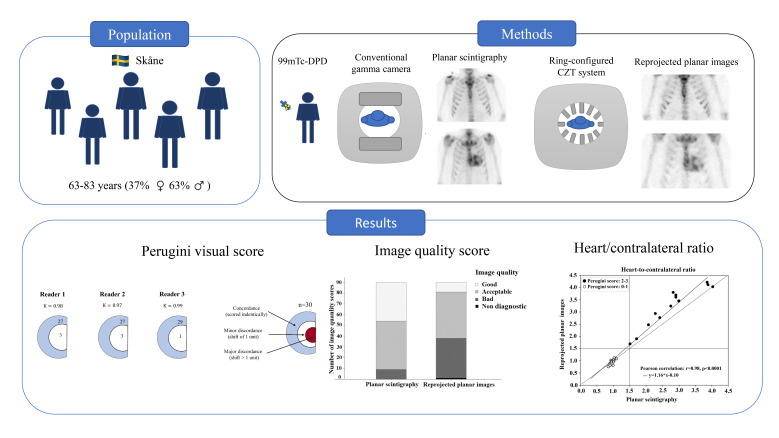

## Introduction

Cardiac amyloidosis is characterized by the accumulation of amyloid fibrils in extracellular tissue. The two main classes of cardiac amyloidosis are transthyretin (ATTR) and light chain (AL) amyloidosis.^1,2^ Scintigraphy with radiopharmaceuticals of phosphate derivatives shows high sensitivity and specificity for diagnosing ATTR amyloidosis, it can distinguish ATTR from AL amyloidosis, and it is widely accepted as a diagnostic tool.^3–7^ The relatively recent expert consensus recommendations for multimodality imaging in cardiac amyloidosis^8,9^ recommend planar scintigraphy with technetium-99m ([^99m^Tc]Tc) phosphonate derivatives combined with single-photon emission computed tomography-computed tomography (SPECT-CT) within 2–3 h post-injection. For assessment of myocardial uptake in planar scintigraphy, the Perugini 4-point visual scoring system (0–3) is recommended.^9^ A Perugini score of 2 or 3 accompanied by the exclusion of plasma cell dyscrasia is indicative of ATTR amyloidosis.^10^ In addition to Perugini visual scoring, semi-quantitative measurements of heart-to-contralateral (H/CL) ratios in planar scintigraphy can be determined, with values ≥1.5 indicating positivity for ATTR amyloidosis.^7,8,11,12^

Until recently, conventional gamma camera systems used for imaging were composed of two detector heads with NaI(Tl) or cadmium zinc telluride (CZT) crystals. In 2018 and 2021, novel ring-configured SPECT-CT systems with CZT detectors were introduced (Spectrum Dynamics Medical Veriton, Caesarea, Israel, and GE Healthcare StarGuide, Haifa, Israel). These ring-configured CZT gamma cameras are SPECT-CT-only systems that are not able to acquire planar scintigraphy. Instead, planar images are reprojected from SPECT-CT data using the reconstructed volume and the attenuation map. The use of reprojected planar images has been validated for conventional NaI(Tl) and dual-head CZT gamma cameras for lung and bone scintigraphy but not for ATTR amyloidosis.^13–15^ The aim of this study was to verify the use of reprojected planar images from a ring-configured CZT gamma camera system in comparison with planar images from a conventional gamma camera for the diagnosis of ATTR amyloidosis. The reprojected images can then be used with the SPECT-CT for a full assessment.

## Methods

### Patient population

The study included a total of 30 patients undergoing clinical evaluation for ATTR amyloidosis and, therefore, was referred for [^99m^Tc]Tc-labelled 3,3-diphosphono-1,2-propanodicarboxylic acid ([^99m^Tc]Tc-DPD) scintigraphy at the Department of Clinical Physiology and Nuclear Medicine, Skåne University Hospital, Sweden. The study was approved by the Swedish Ethical Review Authority (#2020-07213) and was performed in accordance with the Declaration of Helsinki. Written informed consent of the patients was obtained prior to the imaging.

### Image acquisition, camera modality, and image analysis

The patients were injected with 591 ± 14 MBq [^99m^Tc]Tc-DPD and images were acquired 3–4 h post-injection and scanned for one bed position covering the thorax. Imaging was first performed with a conventional gamma camera for 60% of the patients (GE Discovery NMCT 670, GE Healthcare, Milwaukee, WI, USA), followed by a ring-configured CZT gamma camera (GE Healthcare StarGuide). The rest of the patients were first imaged on the ring-configured CZT gamma camera.

When using the conventional gamma camera, planar scintigraphy (anterior) was performed on a dual-head Anger camera with a 3/8-inch NaI(Tl) crystal for 8 min. The patients were scanned with low-energy high-resolution collimators (hole diameter 1.11 mm, septum thickness 0.2 mm, and hole length 40 mm) with a total field of view of 54 cm × 40 cm. The scan was performed with a 256 × 256 matrix and an energy window of 140 keV ± 10%.

When using the ring-configured CZT gamma camera, SPECT imaging was performed for 8 min using bone sweep acquisition. CT was also performed for attenuation correction (AC) (tube voltage 120 kV, automatic tube current 20–90 mA), which covered the same part as the SPECT. The StarGuide system consists of 12 detectors with 7 CZT modules, each resulting in a total axial field of view of 27.5 cm. The system is equipped with tungsten parallel-hole collimators with an energy range of 40–279 keV. The detectors have a sweeping motion, and when combined with gantry rotation, they cover the patient volume within the field of view.^16^

The reconstruction parameters were optimized in a pilot study involving 10 patients. The algorithm used was block sequential regularized expectation maximization (BSREM) with the relative difference prior (RDP) as a regularization method and correction for attenuation, scatter, and resolution recovery.^17,18^ Reconstruction was performed using BySens™ (GE Healthcare StarGuide), which enables the noise-reducing beta parameter to work within an interval by considering attenuation coefficients. The reconstructed SPECT data are then used together with the CT images to create reprojected planar images by transforming the 3D data into a 2D image. Reprojected planar images are then created by integrating the attenuation coefficients along the line of projection from each voxel to the edge of the object. Combining the results into a two-dimensional matrix generates an image that appears to have been scanned from an anterior detector view.

The 10 patients were randomly selected from the total population of 30 patients, and reconstruction was done with beta values of 0.2, 0.3, 0.4, 0.6, and 0.8, a gamma value of 4, and voxel size of 2.46 mm^3^. A 4-point scale was used for visual assessment of image quality in consideration of the noise level, sharpness, and distinction of the ribs (1 = non-diagnostic, 2 = bad image quality, 3 = acceptable image quality, and 4 = good image quality). For assessment of myocardial uptake, a 4-point scale according to the Perugini visual scoring system was used.^10^ The following describes the grading system suggested for interpretation of planar scintigraphy in the expert consensus recommendations: no myocardial uptake and normal bone uptake (Score 0), myocardial uptake less than rib uptake (Score 1), myocardial uptake equal to rib uptake (Score 2), and myocardial uptake greater than rib uptake with mild/absent rib uptake (Score 3).^8,9^ Two experienced nuclear medicine physicians scored the images in a blinded fashion according to the Perugini visual scoring system and the image quality scoring criteria. This procedure was performed to reduce the number of images to assess and optimize image quality. One set of reconstruction parameters was then chosen and applied to all 30 patients’ scans on the ring-configurated CZT gamma camera.

Three experienced nuclear medicine physicians assessed 30 patients (including the 10 patients involved in optimization) using the chosen parameters from the pilot study. In total, 60 images were scored (30 planar from the conventional system and 30 reprojected planar images from the ring-configured CZT system) in a blinded fashion and scored according to the Perugini visual scoring system and the image quality scoring as described above.

Heart-to-contralateral (H/CL) ratios were calculated from the planar scintigraphy obtained with the conventional gamma camera and the reprojected planar images from the ring-configured CZT gamma camera, respectively. A region of interest (ROI) was placed over the heart (a circular ROI with a diameter of 7 cm, including the blood pool, soft tissue, and bone). A contralateral ROI was mirrored from the heart ROI in the right part of the thorax and adjusted to reflect an area equivalent to where the heart ROI was placed (a circular ROI with a diameter of 7 cm including soft tissue and bone). The ratio was calculated as follows: H/CL ratio = (heart ROI mean counts/pixel)/(contralateral ROI mean counts/pixel). An H/CL ratio of ≥1.5 combined with a Perugini visual score of 2–3 suggests an ATTR-positive examination.^7,8,11,12^

### Statistical analysis

Statistical analysis was performed in R (R Core Team, 2023)^19^ and Python (Python Software Foundation, version 2.7).^20^ The level of agreement for each reader was assessed using Cohen’s kappa, and the inter-rater reliability of the three readers was assessed using Fleiss’s kappa. The reported results were the kappa value, 95% confidence interval (CI), and percentage of agreement for Perugini scoring and the image quality scoring. The H/CL ratios were compared using linear regression and a Bland–Altman plot.

## Results

Patient characteristics are presented in *Table 1*.

**Table 1:** Patient characteristics

Patient characteristics	Mean ± SD/%
Age (years)	73 ± 10
Female	11 (37%)
Weight (kg)	82 ± 13
Length (cm)	173 ± 7
BMI (kg/m^2^)	28 ± 4

### Optimization reprojected planar images

**Figure 1:**
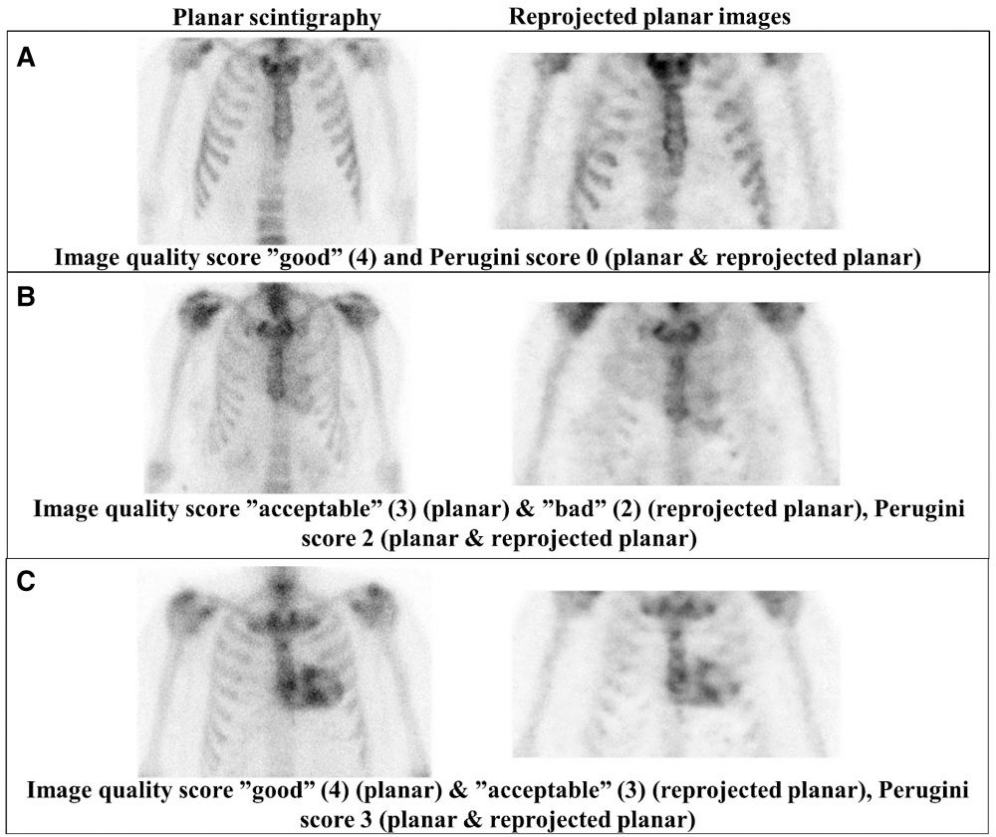
Examples of planar scintigraphy and reprojected planar images from three patients with image quality Score 4 (‘good’) and Perugini visual Score 0 (*A*), image quality Score 3 (‘acceptable’; planar) and Score 2 (‘bad’; reprojected planar), and Perugini visual Score 2 (*B*), and image quality Score 4 (‘good’; planar) and Score 3 (‘acceptable’; reprojected planar) and Perugini visual Score 3 (*C*).

An identical ranking of the Perugini visual score independent of the chosen reconstruction parameters was obtained for the reprojected images of the 10 patients included in the optimization (reconstructed with BSREM and RDP regularizations for beta of 0.2–0.8 in steps of 0.2 and gamma of 4 using BySens, attenuation, and scatter correction and resolution recovery). A similar visual scoring of image quality was also obtained independent of the reconstruction parameters in the reprojected planar images, indicating that the same assessment was obtained within these intervals of the reconstruction. Therefore, one reconstruction alternative was chosen and applied to all 30 patients (BSREM and RDP regularization with beta of 0.4 and gamma of 4 using BySens™, attenuation, and scatter correction and resolution recovery). *Figure 1* shows examples of planar scintigraphy and reprojected planar images, the Perugini visual score, and the image quality score.

### Comparison of planar and reprojected planar images

*Table 2* shows Fleiss’s kappa values, the 95% CIs, and percentages of agreement between the three readers when comparing the Perugini visual score for planar scintigraphy and reprojected planar images. A high level of agreement was obtained for the readers when assessing conventional planar scintigraphy (*κ* = 0.88) and reprojected planar scintigraphy (*κ* = 0.83). These values suggest almost perfect agreement for each method individually.

**Table 2:** Fleiss’s kappa, 95% CI, and percentage agreement for Perugini visual score comparing planar scintigraphy and reprojected planar images for all three readers

	Fleiss’s kappa	95% CI	Percentage agreement (%)
Perugini visual score planar scintigraphy	0.88	0.75–1.00	90
Perugini visual score reprojected planar images	0.82	0.76–1.00	83

**Figure 2:**
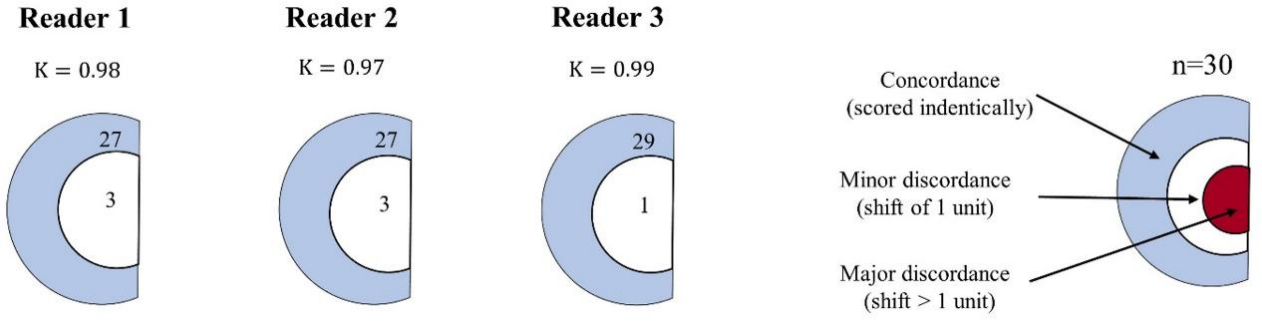
Diagram for comparison of the Perugini visual score for each reader. The number of patients scored in concordance, minor discordance (shift of 1 unit), and major discordance (shift > 1 unit) are presented in semi-circles. For each reader, a kappa (*κ*) value comparing Perugini visual score on planar scintigraphy and on reprojected planar images is presented. Reader 1 (*κ* = 0.98, 95% CI = 0.66–1.01 and percentage of agreement of 90%) and Reader 2 (*κ* = 0.97, 95% CI = 0.67–1.01 and percentage of agreement of 90%) had minor discordance for three patients, Reader 3 (*κ* = 0.99, 95% CI = 0.81–1.05 and percentage of agreement of 97%) had minor discordance for one patient. Reader 1 scored two patients higher and one patient lower in the reprojected planar images. Reader 2 scored all three patients higher in the reprojected images, whereas Reader 3 scored one patient higher in the reprojected planar images.

For the majority of patients, the planar scintigraphy and reprojected planar images Perugini visual scores were concordant. *Figure 2* illustrates the level of concordance and discordance for each reader in the assessment. Reader 1 demonstrated an almost perfect level of agreement (*κ* = 0.98). Out of the 30 patients, 27 were scored in concordance, showing strong alignment in the assessment. For three patients, there was minor discordance with Perugini visual scores of 1 for reprojected planar images and 0 for planar scintigraphy for 2 of these patients. One patient’s Perugini visual score was 0 for the reprojected planar image and 1 for the planar scintigraphy. Similarly, Reader 2 demonstrated an almost perfect level of agreement (*κ* = 0.97). The majority of the patients’ Perugini visual scores (27 out of 30) were concordant. Like Reader 1, Reader 2 also had three cases with minor discordance, and all three had Perugini visual scores of 1 for reprojected planar images and 0 for the planar scintigraphy. Reader 3 had an almost perfect level of agreement (*κ* = 0.99). Scores were concordant for 29 out of 30 patients. Only one patient was scored with minor discordance, where Perugini visual score was 1 for the reprojected planar image and 0 for the planar scintigraphy. For the patients scored with minor discordance, all three readers expressed a desire for SPECT-CT to make an accurate assessment for both the planar scintigraphy and reprojected planar images.

**Figure 3:**
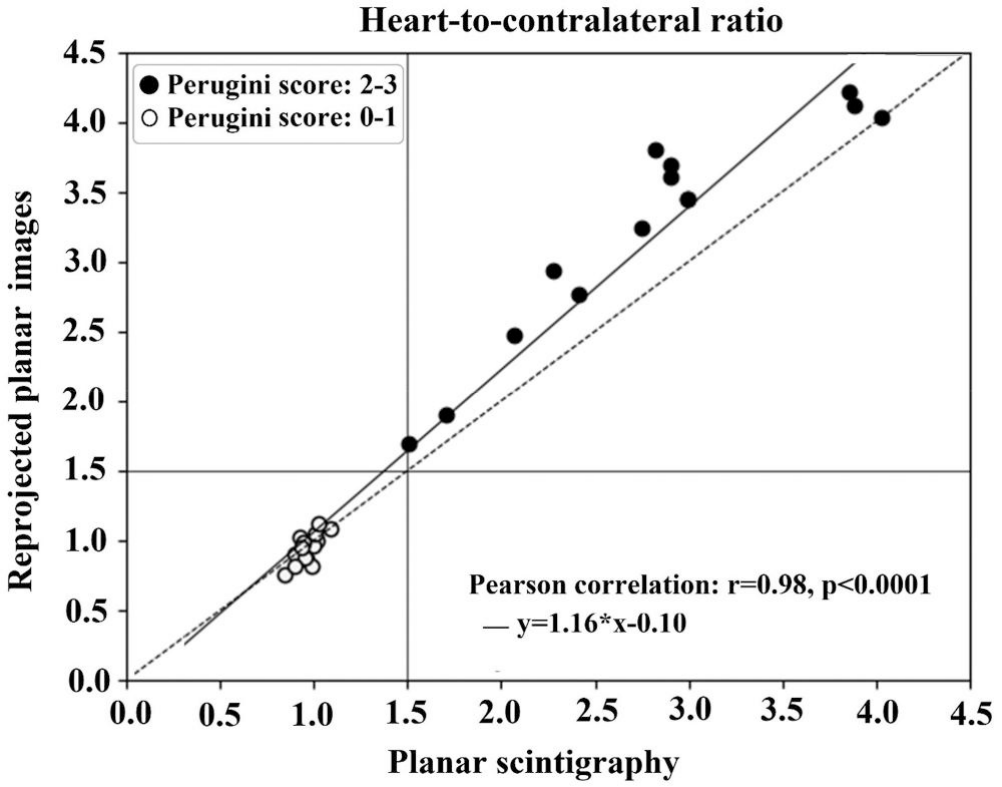
Pearson correlation between H/CL ratios from planar scintigraphy with a conventional gamma camera (*x*-axis) and H/CL ratios from reprojected planar images with a ring-configured CZT gamma camera (*y*-axis). The regression is represented by the solid line, and the identity line is represented by the dashed line. The white data points represent Perugini visual Scores 0–1, and the black data points represent Perugini visual Scores 2–3.

**Figure 4:**
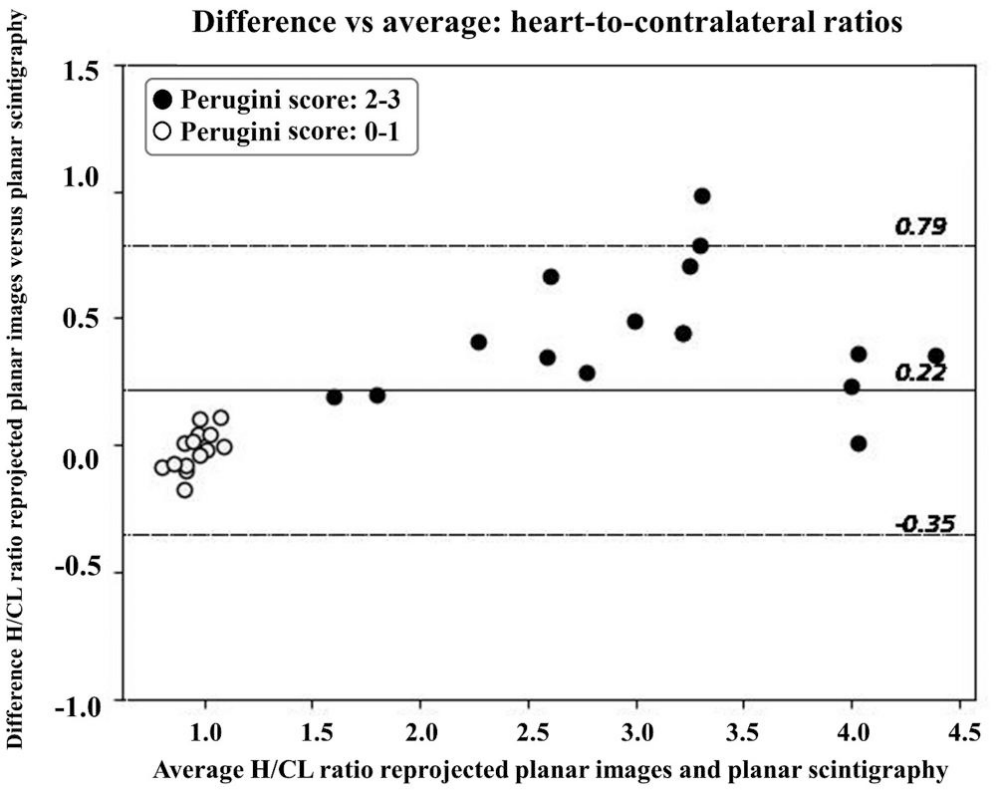
Bland–Altman plot showing the agreement between the average H/CL ratio for reprojected planar images (generated from a ring-configured CZT gamma camera) and planar scintigraphy (acquired with conventional gamma camera) compared with the difference in H/CL ratio between reprojected planar and conventional planar images (mean and 95% limits of agreement are shown). The solid line represents mean bias, and the dashed lines represent limits of agreement. The white data points represent Perugini visual Scores 0–1, and the black data points represent Perugini visual Scores 2–3.

Linear regression and Bland–Altman plots were employed to assess the agreement between the H/CL ratios for the imaging methods (*Figures 3* and *4*). The linear regression analysis showed a strong correlation (*r* = 0.98, *P* < 0.0001). All patients with Perugini visual scores of 0 and 1 (white data points, *Figure 3*) had H/CL ratios <1.5, whereas patients with Perugini visual scores of 2–3 (black data points, *Figure 3*) had H/CL ratios of ≥1.5.

The Bland–Altman plot shows agreement between the H/CL ratios from the planar scintigraphy compared with H/CL ratios from reprojected planar images with a mean difference of 0.22 (95% CI −0.85 to 9.35; *Figure 4*). The majority of the data points were within the limits of agreement, indicating consistency when assessing the ratios between planar scintigraphy and reprojected planar images. For high Perugini visual scores, a trend of larger differences occurred, where reprojected images generated higher H/CL ratios compared with planar scintigraphy. The comparative analysis of H/CL ratios for planar scintigraphy and reprojected planar images revealed a high correlation (*Figure 3*), underscoring a strong linear relationship between the two image sets. However, the Bland–Altman analysis showed that the reprojected planar images generated slightly higher ratios compared with planar scintigraphy, which was particularly pronounced for ratios generated from patients with Perugini score of 3.

**Figure 5:**
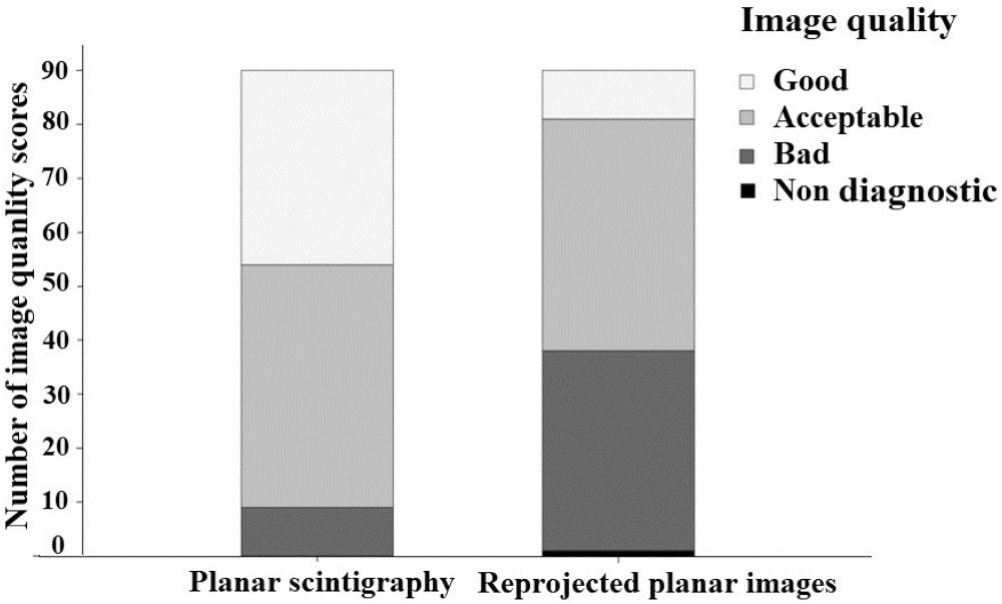
Image quality score for planar images and reprojected planar images. The *y*-axis represents the number of images, 30 planar and 30 reprojected planar, assessed and scored independently by three expert readers resulting in 90 image quality ratings. The figure demonstrates a shift towards lower image quality score for the reprojected planar images compared with the planar images.

*Figure 5* illustrates the image quality scores. Planar scintigraphy was mostly scored as ‘acceptable’ (50%), followed by 40% as ‘good’, and 10% as ‘bad’ (no image was scored ‘non-diagnostic’). Most reprojected planar images were scored as ‘acceptable’ (48%), followed by 41% as ‘bad’. Only 10% of the reprojected planar images were scored as ‘good’. One reprojected planar image was scored as ‘non-diagnostic’ according to one reader, while the other two readers scored the image quality as bad. Thus, for image quality, a shift towards lower image quality was seen for the reprojected planar images compared with the planar scintigraphy.

## Discussion

A ring-configured CZT gamma camera does not allow for planar scintigraphy. This study verifies the use of reprojected planar images as a substitute for planar scintigraphy to comply with expert consensus recommendations that advocate for the combined use of planar and SPECT-CT scintigraphy when diagnosing ATTR amyloidosis. This is the first study exploring the possibility of using reprojected planar images generated from a ring-configured CZT gamma camera when assessing ATTR amyloidosis with [^99m^Tc]Tc-DPD scintigraphy. The results showed a strong level of agreement in the diagnostic Perugini visual scores. In addition to the visual assessment of myocardial uptake, a calculation of H/CL ratio was also performed, showing a strong level of agreement between the reprojected planar images and planar scintigraphy from a conventional gamma camera, also supporting the well-established cut-off value of 1.5.^7,8,11,12^ However, there was a shift towards lower image quality for the reprojected planar images compared with planar scintigraphy.

Bailey *et al*.^14^ compared the use of reprojected planar images generated from SPECT using a synthetic attenuation map for AC with conventional planar scintigraphy for ventilation/perfusion SPECT. The reprojected planar images had the same diagnostic sensitivity as planar scintigraphy. Harris *et al*.^21^ studied the use of reprojected planar images for diagnosing pulmonary embolism and also reported strong agreement with planar scintigraphy. The comparison of reprojected planar images and planar scintigraphy for bone scans was studied by Arvola *et al.*,^13^ reporting excellent sensitivity in the reprojected planar images. Bailey *et al*., Harris *et al*., and Arvola *et al*. all reported an increased detection of defects/lesions in reprojected planar images.^21^ However, for bone scans, Arvola *et al.* reported that lesions rated as true positive turned out to be false positive for three patients when compared with magnetic resonance imaging, fluorine 18-sodium fluoride positron emission tomography-computed tomography (^18^F-NaF PET-CT), and ^18^F-prostate-specific membrane antigen PET-CT (^18^F-PSMA-1007 PET-CT), suggesting that reprojected images should not be used exclusively, but instead should be used in conjunction with SPECT-CT for more accurate assessments.^13^

Our results show a shift towards lower image quality for the reprojected planar images compared with conventional planar scintigraphy. This is also in line with results reported by Arvola *et al*.^13^ where image quality scores for conventional planar scintigraphy were generally higher. Since physicians are more experienced in reading planar scintigraphy images, some variations in the visual image quality are anticipated as a contributing factor for the lower image quality score in reprojected planar images. An increase in image quality would be expected in reprojected planar images because they are generated from reconstructed SPECT-CT data that are corrected for attenuation, scatter, and resolution recovery, all of which affect the visual impression of a nuclear medicine image. However, comparing the ribs in the planar scintigraphy and reprojected planar images in *Figure 1B* and *C*, the uptake in the reprojected images looks somewhat inhomogeneous, and it is harder to distinguish the shape of the ribs. The inhomogeneous appearance in the reprojected planar images may be caused by the Bayesian reconstruction method, which is used to reconstruct the reprojected planar images in this study.

It has been shown that ring-configured CZT gamma cameras using Bayesian reconstruction methods have superior sensitivity and higher energy resolution compared with a conventional gamma camera and that the image quality can be maintained with half the acquisition time.^16,22,23^ In this study, the improved energy resolution and sensitivity were not studied specifically, but the acquisition of [^99m^Tc]Tc-DPD scintigraphy can be further optimized with this knowledge. By reducing the energy window width, scattered photons would be excluded, which could contribute to improving image resolution. The scan time on the ring-configured CZT gamma camera is 8 min in this study. However, there is potential for further optimization, as it has been demonstrated that a good image quality can be achieved with a shorter acquisition time for ring-configured CZT gamma cameras, e.g. performing a bone scan with SPECT-CT for 4 min per bed position (BP) has been shown to give equivalent image quality as performing the scan for 8 min/BP.^16^

An advantage of generating reprojected planar images on a ring-configured CZT gamma camera is that the separate planar acquisition is eliminated, allowing for a significant reduction in the total scan time. A reduced scan time could potentially increase patient comfort, especially for patients who have a hard time lying still during a long acquisition. Furthermore, a reduced scan time could decrease the risk of image artefacts due to patient motion during image acquisition and enables higher patient throughput. This study has resulted in a transition from the use of planar scintigraphy along with SPECT-CT to SPECT-CT-only acquisition using reprojected planar images for both conventional gamma cameras and ring-configured CZT gamma cameras at our department.

Overall, a high linear correction is obtained for H/CL ratios, showing a strong positive relationship between planar scintigraphy and reprojected planar images. The Bland–Altman plot in *Figure 4* indicates that for high H/CL ratios (Perugini visual Score 3), a proportional bias is introduced in the discrepancy between planar scintigraphy and reprojected planar images. The reprojected planar images generate a higher H/CL ratio for values corresponding to visual Perugini Score 3, suggesting that the relationship is not constant across all levels of measurements, which could be scale dependency or non-linearity for higher H/CL values. A possible reason might be the insufficient implementation of correction techniques, such as effects like the tailing phenomenon in the scatter correction, which is not corrected for in the scatter compensation for the ring-configured CZT system.^24^ Therefore, studies with a larger number of patients are needed for a more accurate analysis of the proportional bias indicated for high H/CL ratios observed for reprojected planar images generated from ring-configured CZT gamma cameras. However, since an even higher H/CL ratio would not be evaluated differently, the clinical outcome would not be affected. Another reason to study a larger patient population is to gain clearer insights into borderline cases. Rating is somewhat more difficult for borderline uptake when the Perugini visual score is between 1 and 2.^25^

One limitation in this study is that SPECT-CT was not included in the assessment. SPECT-CT is a part of routine assessment for [^99m^Tc]Tc-DPD scintigraphy for ATTR amyloidosis. In this study, SPECT-CT images were not acquired with both the conventional gamma camera and the ring-configured CZT gamma camera, since the aim of this study was to verify the use of reprojected planar images due to the patient population and their general state of health. For full assessment, SPECT-CT should serve as a complement to reprojected planar images, preventing misinterpretation of distinguishing between Perugini visual Scores 0 and 1. Two other studies show that SPECT-CT can outperform planar scintigraphy when assessing ATTR amyloidosis, which raises the question of whether planar scintigraphy is needed at all.^26,27^ If SPECT-CT images are used for visual assessment only, other reconstruction parameters may be more suitable than the ones presented in this paper since they are optimized for reprojected planar images. The use of prior terms in the reconstruction process is relatively new for SPECT-CT imaging and further assessment of optimal reconstruction parameters for SPECT-CT assessment may be warranted.^16,17,28,29^ Therefore, it is of further interest to verify assessments using only SPECT-CT and no planar scintigraphy or reprojected planar images.

SPECT-CT-only assessment for diagnosing ATTR amyloidosis is of interest due to the potential advantage of excluding false positives and the possibility of performing quantitative imaging. However, to do this, both Perugini visual score and H/CL ratios need to be verified on the SPECT-CT images. To the best of our knowledge, this has not been done yet which means that both of these scoring systems rely primarily on planar scintigraphy. Verifying reprojected images created from SPECT-CT would be the first step.

## Conclusion

A ring-configured CZT gamma camera can be used for diagnosing ATTR amyloidosis. By reprojecting planar images and using SPECT-CT as a complement, both Perugini visual score and H/CL ratio can be used to correctly assess and diagnose ATTR amyloidosis in accordance with expert consensus recommendations.

## Data Availability

The data will be made available upon appropriate request from the authors.

## Lead author biography

Irma Cerić Andelius is a medical physicist at Skåne University Hospital, Sweden. Her clinical work and research involve the implementation of new camera techniques in clinical routine, as well as the continuous improvement and further development of current methods. She focuses on CZT detectors to evaluate their potential in improving outcomes, with a particular emphasis on new architecture and improved reconstruction algorithms to enhance image quality.
